# A novel autophagy-related genes prognostic risk model and validation of autophagy-related oncogene VPS35 in breast cancer

**DOI:** 10.1186/s12935-021-01970-4

**Published:** 2021-05-17

**Authors:** Xiaoying Li, Yu Cao, Xinmiao Yu, Feng Jin, Yang Li

**Affiliations:** 1 Department of Breast Surgery, The First Affiliated Hospital of China Medical University, 155 Nanjing Road, Shenyang, 110001 China; 2 Department of Cell Biology, Key Laboratory of Cell Biology, National Health Commission of the PRC, and Key Laboratory of Medical Cell Biology, Ministry of Education of the PRC, China Medical University, No. 77, Puhe Road, Shenyang North New Area, Shenyang, 110122 Liaoning China

**Keywords:** Breast cancer, Autophagy, Autophagy-related encoding genes (ARGs) prognosis, Risk model, VPS35

## Abstract

**Background:**

Accumulating evidence implies that autophagy plays a critical role in breast cancer development and progression. It is crucial to screen out autophagy-related encoding genes (ARGs) with prognostic value in breast cancer and reveal their biological properties in the aggressiveness of breast cancer.

**Methods:**

Univariate and multivariate Cox proportional hazards analyses were used to identify a prognostic risk model of ARGs from The Cancer Genome Atlas (TCGA). Kaplan–Meier analysis, univariate and multivariate Cox regression analyses and receiver operating characteristic (ROC) curve analysis were performed to validate the risk model. Western blot and immunohistochemistry (IHC) were conducted to assess the expression of VPS35 (one of ARGs in risk model). CCK8, Colony formation assay, Transwell migration/invasion assays and autophagy flux assay were used to confirm biological function of VPS35 in breast cancer.

**Results:**

In this study, the prognostic risk model consisting of six ARGs (VPS35, TRIM21, PRKAB2, RUFY4, MAP1LC3A and LARP1) in breast cancer were identified. The risk model was further verified as a novel independent prognostic factor for breast cancer patients. We also clarified that vacuolar protein sorting-associated protein 35 (VPS35), one of ARGs in the risk model, was upregulated in breast cancer samples and cell lines. VPS35 overexpression was correlated with more aggressive phenotype of breast cancer and indicated worse prognosis in both progression-free survival and overall survival analyses. Meanwhile, VPS35 knockdown inhibited breast cancer cell proliferation, migration and invasion, suggesting that VPS35 promoted the progression of breast cancer. VPS35 silence also influenced autophagy process, indicating that VPS35 was essential for autophagy completion.

**Conclusion:**

Taken together, the six ARGs risk model has a remarkably prognostic value for breast cancer. Among them, VPS35 might exert as a significant oncogenic and prognostic factor for breast cancer and could be a promising autophagy-related therapeutic target in clinical practice.

**Supplementary Information:**

The online version contains supplementary material available at 10.1186/s12935-021-01970-4.

## Background

Breast cancer is the most frequent malignancy and the leading cause of cancer-associated mortality in women worldwide [1, 2]. With the advent of the era of precision medicine, individual specific targeted therapy has aroused broad concern in clinical practice [3]. Thus, exploring potential prognostic biomarkers and promising specific targets is considered to be a crucial step to achieve this process.

Autophagy is well known for its important role in sustaining cellular homeostasis via a series of degradation processes [4]. Autophagy is implicated in many vital biological processes including stress and starvation adaptation, metabolism to maintain stable intracellular environment [5, 6]. Besides, autophagy also has critical effects in various human diseases, such as inflammation, neurodegenerative disorders and cancer [7, 8]. During the past few years, accumulating evidences have suggested that autophagy is involved in breast cancer development and aggressiveness [9, 10]. Recently, more and more researches have indicated that autophagy-related encoding genes influence cancer progression and survival prognosis [11–13]. Therefore, identifying essential autophagy-related encoding genes (ARGs) closely associated with prognosis in breast cancer is of great significance for both theoretical foundation and clinical guidance.

In this study, we investigated autophagy-related encoding genes expression in breast cancer from The Cancer Genome Atlas (TCGA). We finally obtained a six ARGs signature with prognostic value in breast cancer patients. Among the six ARGs, VPS35 was a high-risk factor for prognosis of breast cancer. Nonetheless, little is known about the association of VPS35 with cancer and there are no reports regarding the relationship between VPS35 and breast cancer up to now.

Herein, we investigated VPS35 expression status in breast cancer specimens and firstly assessed the correlation of VPS35 with clinical pathological factors and survival prognosis in breast cancer. We further confirmed the oncogenic role and function of VPS35 in breast cancer progression. This study might provide theoretical basis for finding novel autophagy-related prognostic biomarkers and therapeutic targets of breast cancer.

## Materials and methods

### Patient data sets

Breast cancer patients with clinical information and pathology records were obtained from the TCGA (https://cancergenome.nih.gov/). Normalize gene expression was performed by the edgeR package. In this study, a total of 1053 TCGA female breast cancer patients with encoding gene expression profiles were used. Among them, 986 patients with complete follow-up information and survival time ≥ 30 days and 539 patients with complete clinicopathological data were selected into subsequent analyses. The clinical features are detailed in Table 1 [14].

**Table 1:** Clinical pathological parameters of patients with breast cancer from TCGA

Feature	N (539)	%
Age (years)		
> 60	227	42.1
≤ 60	312	57.9
T classification		
T1 (< 2 cm)	147	27.3
T2 (2–5 cm)	323	59.9
T3 (≥ 5 cm)	55	10.2
T4 (chest wall and/or skin invasion)	14	2.6
N classification (pN)		
N0 (no metastasis)	259	48.1
N1 (1–3 metastasis)	178	33
N2 (4–9 metastasis)	64	11.9
N3 (≥ 10 metastasis)	38	7
M classification		
M0 (no distant metastasis)	528	98
M1 (distant metastasis)	11	2
TNM stage		
I	96	17.8
II	318	59
III	114	21.2
IV	11	2
ER		
Negative	127	23.6
Positive	412	76.4
PR		
Negative	175	32.5
Positive	364	67.5
HER2		
Negative	440	81.6
Positive	99	18.4
Molecular subtypes		
HER2 amplification	92	17.1
Luminal A/B	419	77.7
TNBC	28	5.2

*T* tumor size, *N* lymph node, *M* distant metastasis, *TNM stage* according to AJCC 8th classification, *TNBC* triple-negative breast cancer

### Identification of autophagy-related encoding genes in breast cancer

A total of 395 autophagy-related encoding genes (ARGs) were extracted from the Molecular Signatures Database of Gene Set Enrichment Analysis (GSEA: M27935, M6328 and M10281).

### Identification of autophagy-related prognostic signatures for breast cancer

To identify ARGs associated with survival, we performed univariate Cox proportional hazards analysis according to the criteria of p < 0.01. Subsequently, multivariate Cox analysis was conducted to construct the optimal prognostic risk model based on the Akaike information criterion (AIC = 1480.25), using the survival R package. Based on the following formula, the risk score for each patient was calculated.

${\text{Risk}}\;{\text{score}} = {\text{coef}}\left( {{\text{mRNA1}}} \right) \times {\text{expr}}\left( {{\text{mRNA1}}} \right) + {\text{coef}}\left( {{\text{mRNA2}}} \right) \times {\text{expr}}\left( {{\text{mRNA2}}} \right) + \cdots + {\text{coef}}\left( {{\text{mRNAn}}} \right) \times {\text{expr}}\left( {{\text{mRNAn}}} \right)$coef (mRNAn) was defined as the coefficient of encoding genes correlated with survival. expr (mRNAn) was defined as the expression of encoding genes.

Based on the median risk score, breast cancer patients in the TCGA were divided into a high-risk group and a low-risk group. Kaplan–Meier survival analysis was performed to estimate the survival difference between the two groups by using the survival and survminer R packages.

### Independent prognostic analysis and ROC curve plotting

To assess the relationship of survival prognosis with clinicopathological factors and risk score, we performed Univariate and multivariate Cox regression analyses using the Survival R package. Time-dependent receiver operating characteristic (ROC) curves were drew to estimate the predictive accuracy for survival time by different clinical pathological factors and risk score using the survival ROC R package.

### Patient specimens

All patients were diagnosed with invasive ductal breast carcinoma in the First Affiliated Hospital of China Medical University. None of the patients had received preoperational radiotherapy or chemotherapy. Breast cancer specimens (n=120 patients) were obtained from patients hospitalized between October 2015 and October 2016.

The deadline date of follow-up was October 2020. All 120 patients had a definite histological pathological diagnosis of breast cancer according to the American Joint Committee Cancer (AJCC) standard. The average age of the 120 patients was 52 years.

Fresh tumor and adjacent noncancerous tissues were collected at the time of surgical resection and immediately stored in liquid nitrogen until protein extraction for Western blot. This study was approved by Ethics Committee of China Medical University.

### Immunohistochemistry (IHC)

IHC was performed as previously described [15]. Briefly, sections from paraffin embedded tumor tissues from patients underwent surgical dissections with VPS35 antibody (Abcam, ab157220; 1/100 dilution). Results were evaluated by two pathologists who were blinded to the experiment separately. VPS35 immunoreactivity was quantified using a combined “H score”, which assesses both the staining intensity and (0, negative; 1, weak; 2, moderate; 3, strong) and the percentage of cells positively stained (0, <5%; 1, 5–25%; 2, 26–50%; 3, 51–75%; 4, 76–100%). Scores of more than or equal to 4 were defined as positive expression.

### Cell culture and lentiviral infection

Breast cancer cell lines MDA- MB-231 and SK-BR-3 were obtained from ATCC (Manassas, VA, USA). MDA-MB-231 cells were cultured in Leibovitz's L-15 (Gibco) with 10% FBS (Gibco). SK-BR-3 cells were cultured in McCoy’s 5A (Gibco) with 10% FBS (Gibco). All cells were incubated in a 5% CO_2_ air at 37 °C. The shRNA expressing lentivirus for VPS35 was purchased from Beijing Syngentech Co., LTD. The VPS35 shRNA 1# sequence was 5′‐GGAGGTCTACCTGACAGATGA‐3′; the VPS35‐shRNA 2# sequence was 5′‐GGTCTGTTTCTTCGAAATTAC‐3′; the VPS35‐shRNA 3# sequence was 5′‐GCAGGAAATGCATCACAATTA‐3′ and the shRNA control sequence was 5′‐AAACGTGACACGTTCGGAGAA‐3′. MDA‐MB‐231 and SK-BR-3 cells were seeded into 12‐well plates overnight. Then, the cells were infected with VPS35‐shRNA 1#, VPS35‐shRNA 2#, VPS35‐shRNA 3# and control lentivirus following the manufacturer's guidelines (Beijing Syngentech Co., LTD.); 5 μg/ml puromycin (Sigma) was added to the medium to select infected cells.

### Western blot

In brief, cells were lysed with RIPA buffer containing 1% protease inhibitor cocktail (Roche, Germany) on ice for 30 min and then lysates were centrifuged. Protein concentrations were measured using the BCA assay kit (KeyGen). Cell lysates were separated by SDS-PAGE and transferred to poly-vinylidene fluoride membranes (Millipore, Billerica, MA, USA) and the membranes were incubated with primary antibodies overnight at 4 °C. The primary antibody used in western blot was anti-VPS35 (Abcam, ab157220; 1/10000 dilution). All western blots were derived from the same experiment and were processed in parallel.

### CCK8 proliferation assay

In brief, shVPS35 and nonspecific control (Con) stable transfected MDA‐MB‐231 cells were seeded in 96‐well plates (3×10^3^) for cell viability assay. CCK8 reagent was added to incubate at 37 °C for 2h. The data was calculated according to the reagent instructions. The absorbance of each sample was measured at 450 nm.

### Colony formation assay

For Colony formation assay, 5×10^2^ MDA-MB-231 cells were seeded into 6‐well plates and cultured at 37 °C in 5% CO2 for 14 days. Then, the cells are fixed and stained, and the number of colonies was measured.

### Transwell migration and invasion assays

For Transwell migration assay, shVPS35 and Con lentivirus‐infected MDA‐MB‐231 cells (2×10^4^ cells in 100 μl Leibovitz's L-15) were separately placed in the top chamber of transwell chambers (8‐μm BioCoat Control Inserts, Corning Costar). The lower chamber was filled with 600 μl Leibovitz’s L-15 supplemented with 10% FBS. After 24 hours incubation at 37 °C, the cells were fixed and stained. The cells in the top chambers were removed with cotton swabs very carefully and counted (five random fields per well at 100× magnification) under a light microscope. For invasion assay, 3×10^4^ cells were plated in the matrigel‐coated chamber and the migration assay was performed.

### Autophagy flux

HEK293 Cells infected with Ad-mCherry-GFP-LC3 (Hanbio Biotech) were seeded into 12-well plates and infected with shVPS35 or Con lentivirus. After 48 h, the cells were fixed with cold methanol and permeabilized with 1‰Triton X-100 in PBS for 15 min.

### Statistical analysis

All statistical analyses were performed using R software (version 3.6.2). The correlation between 6 autophagy-related proteins expressions and clinicopathological factors was analyzed by ggpubr R package. Statistical analyses were conducted using SPSS 20.0 (Chicago, IL, USA) and GraphPad Prism 8.0 software. All data are presented as the means ± standard deviations (SD) and are representative of at least three experiments. Two-sided Student’s t-test was performed between two groups. *p < 0.05, **p < 0.01, ***p < 0.001 and ****p < 0.0001 were considered statistically significant.

## Results

### Identification of autophagy-related encoding genes with significant prognostic value in breast cancer

**Fig. 1:**
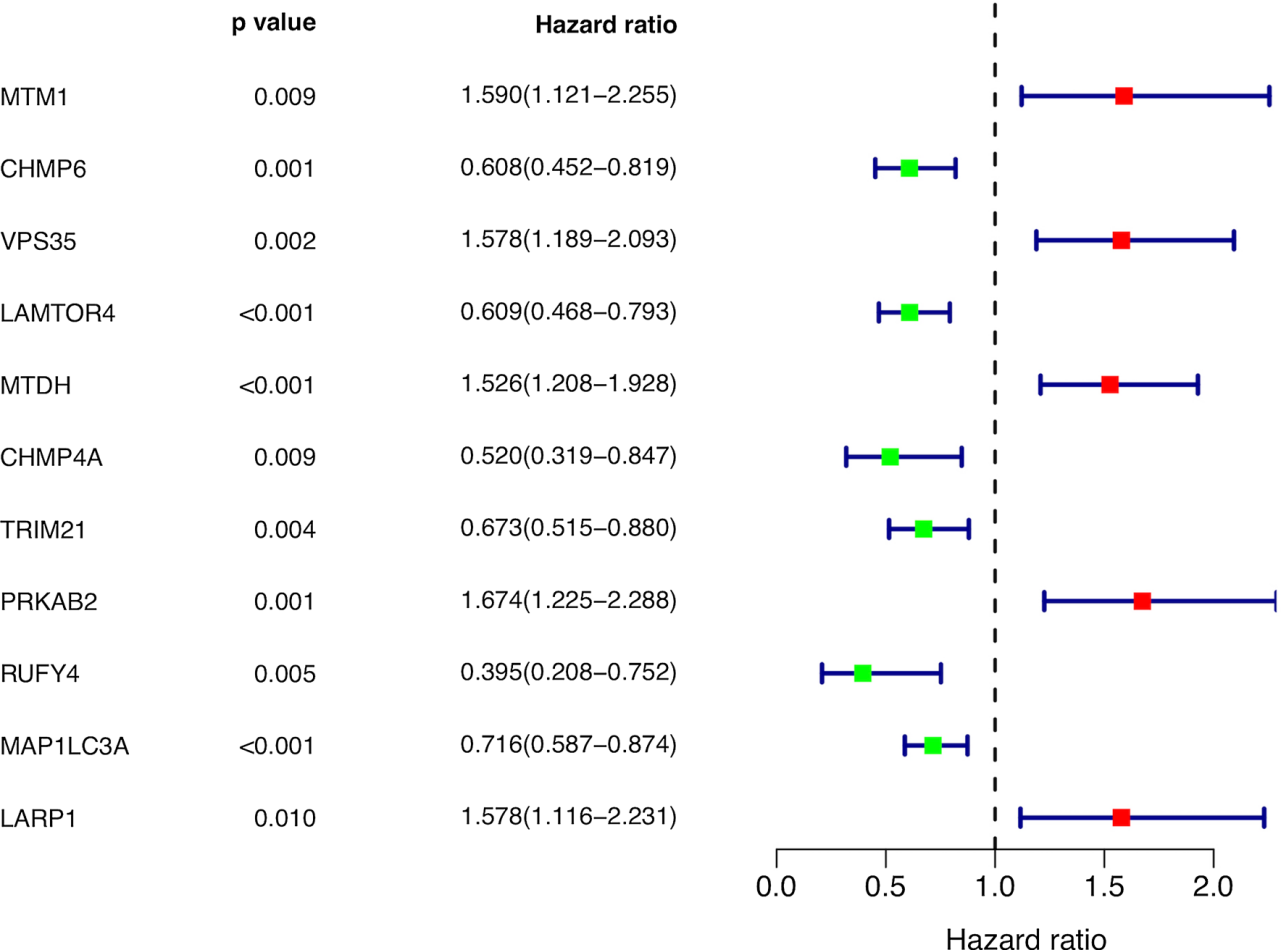
Identification of autophagy-related encoding genes with significant prognostic value in breast cancer by univariate Cox proportional hazards analysis

**Fig. 2:**
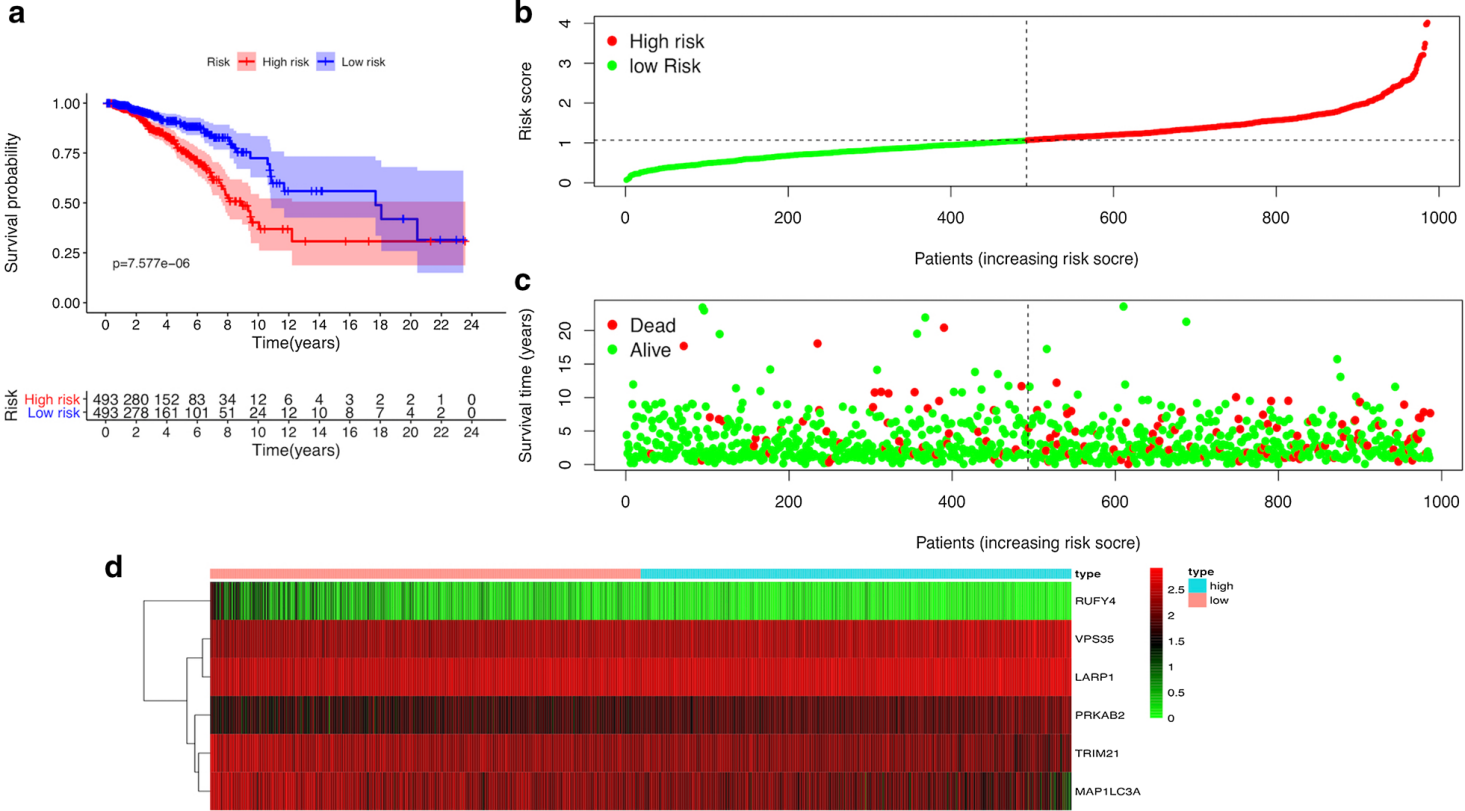
The prognostic value of the risk model of the six autophagy-related encoding genes in the TCGA cohort. **a** Kaplan–Meier survival analysis of the high-risk and low-risk groups based on the risk model and median risk score. **b** The risk curve based on the risk score of each sample. **c** The scatterplot based on the survival status of each sample. The green and red dots represent survival and death, respectively. **d** The heatmap displayed the expression levels of autophagy-related encoding genes in the high-risk and low-risk groups

A total of 11 autophagy-related encoding genes (ARGs) were significantly associated with the survival of breast cancer patients from the TCGA (p < 0.01) by Cox proportional-hazards analysis, including 6 genes with low risk (hazard ration (HR) < 1) and 5 genes with high risk (hazard ration (HR) > 1) (Fig. 1). Subsequently, multivariate Cox analysis further screened 6 genes from the above 11 ARGs with prognostic significance, namely, VPS35, TRIM21, PRKAB2, RUFY4, MAP1LC3A and LARP1 (Table 2). These 6 genes established the optimal autophagy-related prognostic risk model. Breast cancer patients were divided into a high-risk group and a low-risk group based on the median risk score calculated by the risk score formula. The overall survival (OS) of high-risk group was worse than the low-risk group by Kaplan–Meier survival analysis (p = 7.577e−06) (Fig. 2a), suggested that the risk score has prognostic value. The risk curve and scatterplot results showed that the risk score contribute to predict the occurrence of breast cancer mortality (Fig. 2b, c). The heatmap displayed that VPS35, PRKAB2 and LARP1 were highly expressed in the high-risk group, while TRIM21, RUFY4 and MAP1LC3A were upregulated in the low-risk group (Fig. 2d). Based on the above, these 6 ARGs with prognostic significance had been identified in breast cancer.

**Table 2:** The risk model of six autophagy-related encoding genes with prognostic value for breast cancer by multivariate Cox proportional hazards analysis

Gene	Coef	HR	HR.95L	HR.95H	p value	Risk
VPS35	0.278	1.32	0.976	1.786	0.071	High
TRIM21	− 0.228	0.796	0.584	1.085	0.149	Low
PRKAB2	0.255	1.291	0.92	1.811	0.14	High
RUFY4	− 0.606	0.545	0.27	1.102	0.091	Low
MAP1LC3A	− 0.189	0.828	0.666	1.029	0.089	Low
LARP1	0.314	1.369	0.938	1.999	0.103	High

*coef* the coefficient of genes correlated with surviva, *HR* hazard ratio, *HR.95L* low 95%CI of HR, *HR.95H* high 95%CI of HR

### Evaluation of the risk model of 6 autophagy-related encoding genes as an independent prognostic factor for breast cancer patients

**Fig. 3:**
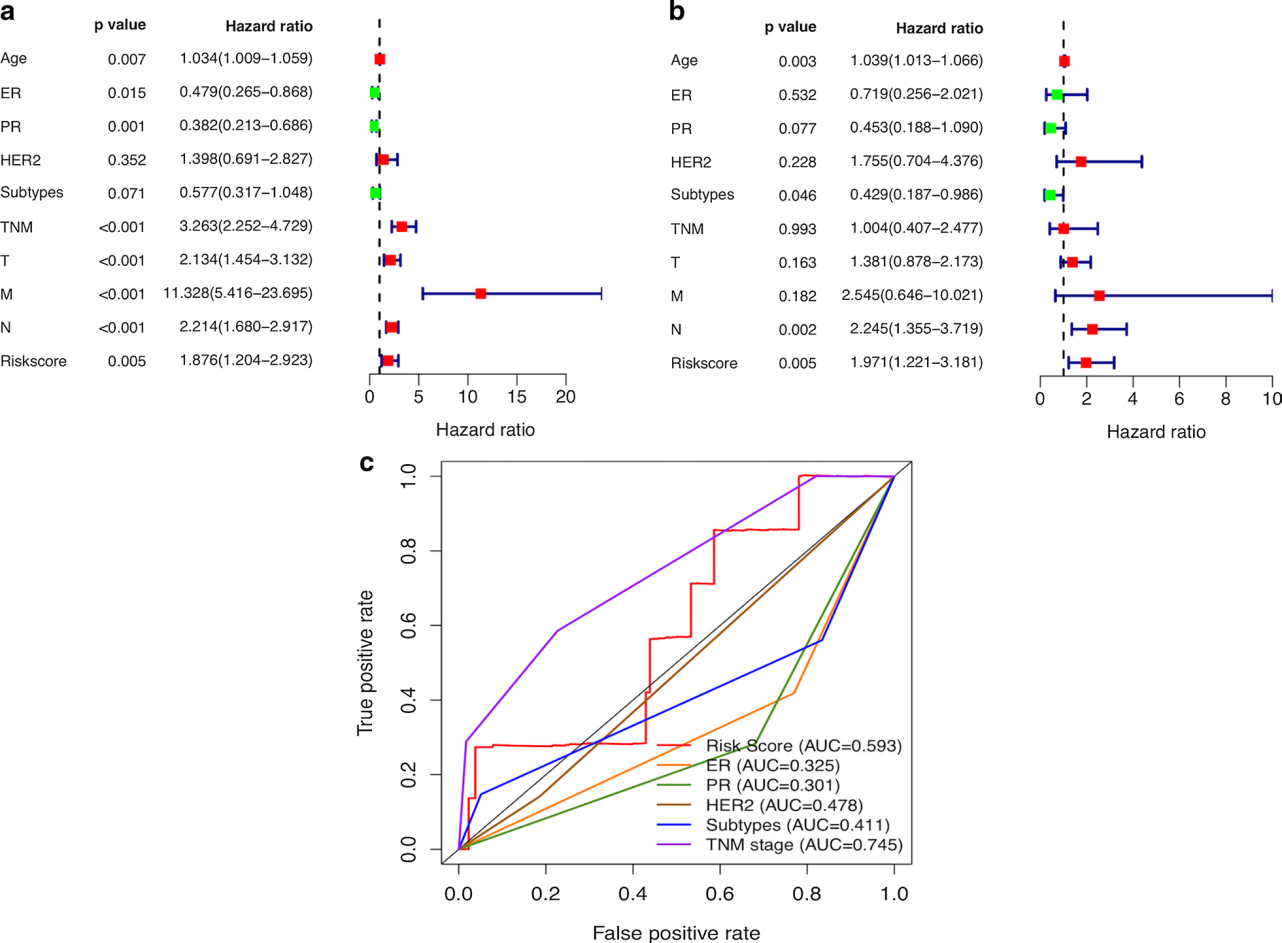
Assessment of the prognostic risk model of the six autophagy-related encoding genes in breast cancer. **a**, **b** The univariate and multivariate Cox regression analysis of risk model score and clinical features regarding prognostic value. **c** The AUC for risk model score and clinical features according to the ROC curves. Clinical features: Age, ER, PR, HER2, Subtypes (molecular subtypes), TNM, T (tumor size), N (lymph node metastasis) and M (distant metastasis)

In order to clarify whether the autophagy-related risk model is an independent prognostic factor for breast cancer, univariate and multivariate Cox regression analyses were performed. Univariate and multivariate Cox regression analyses showed that the hazard ratio (HR) of the risk score and 95% CI were 1.876 and 1.204–2.923 (p = 0.005), and 1.971 and 1.221–3.181 (p = 0.005), respectively (Fig. 3a, b). These indicated that the autophagy-related risk model has prognostic significance for breast cancer, independent of clinicopathological parameters. The area under the ROC curve (AUC) of the risk score was calculated to assess the sensitivity and specificity in predicting prognosis of breast cancer patients. The AUC of the risk score was 0.593 (Fig. 3c), indicating that the prognostic risk model is considerably reliable. Taken together, these all elucidated that the autophagy-related risk model has become a novel independent prognostic factor for breast cancer patients.

### Correlation of the expression of the 6 autophagy-related encoding genes with clinicopathological factors

**Fig. 4:**
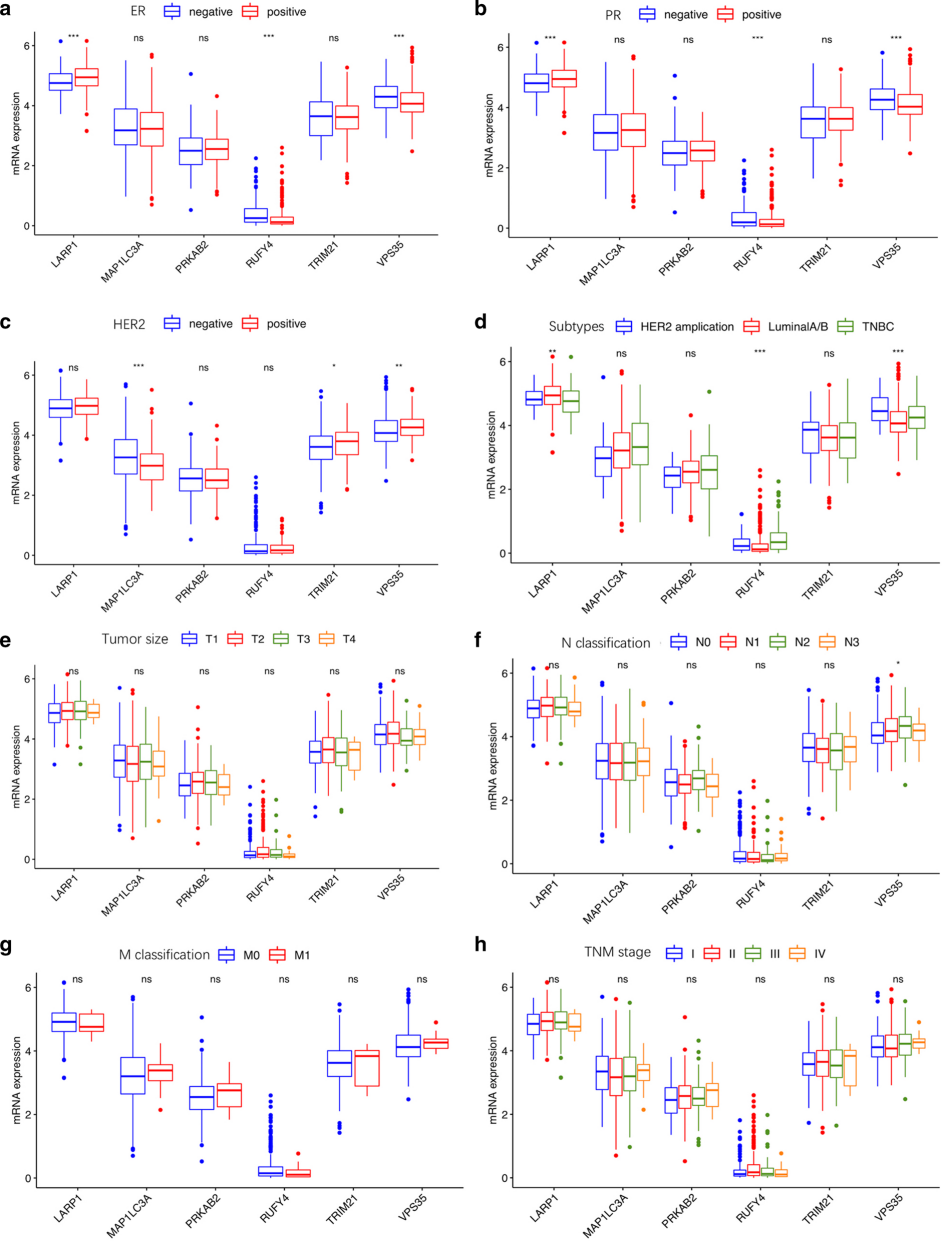
The correlation of the expression of the six autophagy-related encoding genes with clinicopathological factors. **a** ER expression. **b** PR expression. **c** HER2 expression. **d** Subtypes (LuminalA/B; HER2 amplification; TNBC: triple-negative breast cancer). **e** TNM stage. **f** Tumor size (T1: < 2 cm; T2: ≥ 2 cm and < 5 cm; T3: ≥ 5 cm; T4: invasion of chest wall and/or skin). **g** N classification (N0: no lymph node metastasis; N1: 1–3 lymph node metastasis; N2: 4–9 lymph node metastasis; N3: ≥ 10 lymph node metastasis). **h** M classification (M0: no distant metastasis; M1: distant metastasis). ns: no statistical significance, *p < 0.05, ** *p* < 0.01, *** *p* < 0.001, **** *p* < 0.0001

To further assess whether the 6 autophagy-related encoding genes participated in the development of breast cancer, we investigated the association of their expressions with clinicopathological factors. There were remarkably correlations between VPS35 and ER (estrogen receptor)/PR (progesterone receptor) negative, lymph metastasis, HER2 (ERBB2 receptor) positive and triple negative molecular subtypes (ER, PR and HER2 negative), as shown in Fig. 4.

### High expression levels of VPS35 proteins are associated with clinical pathological factors and poor clinical outcomes of breast cancer patients

**Fig. 5:**
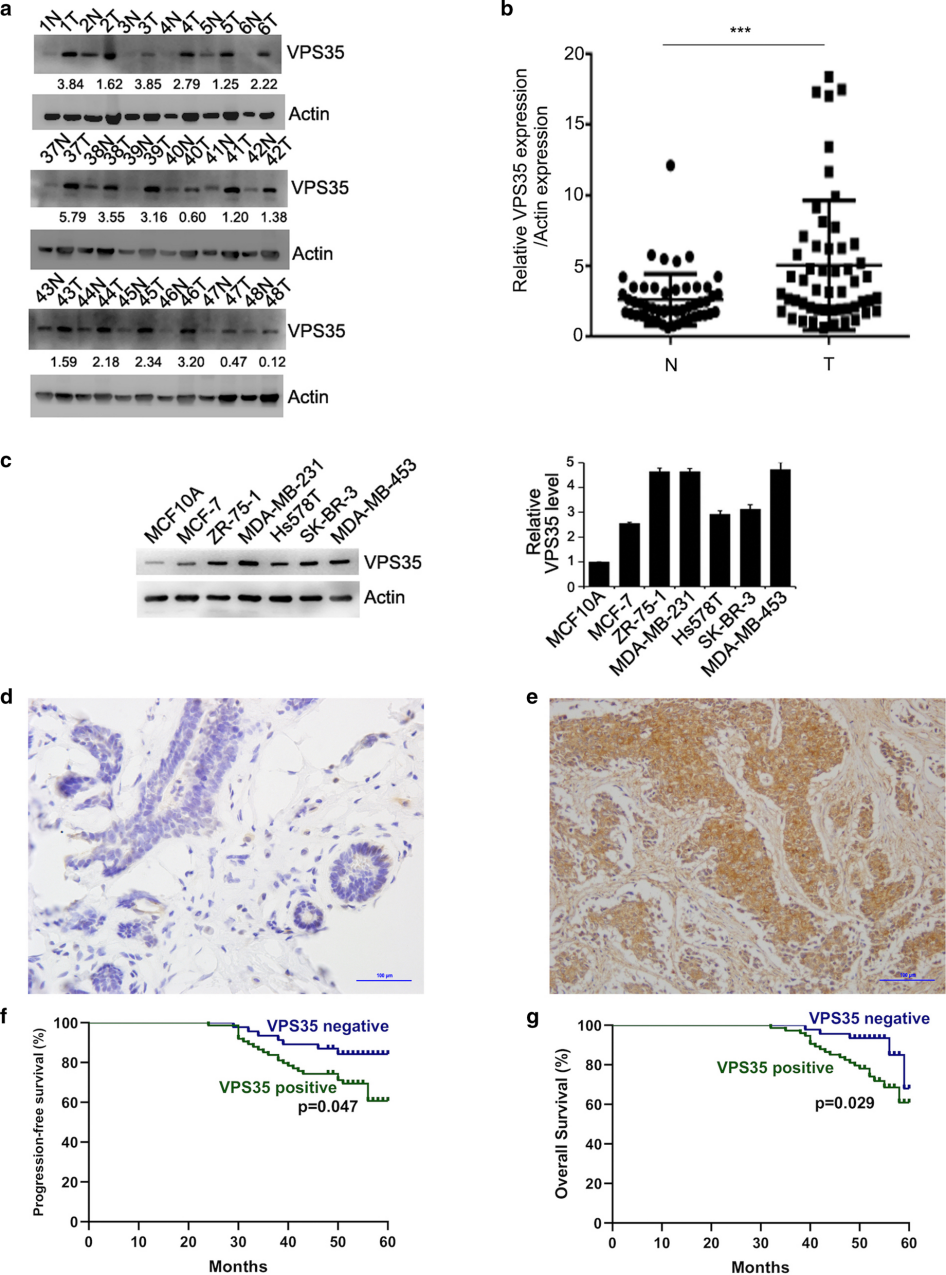
VPS35 expression is upregulated in breast cancer and high expression levels of VPS35 proteins are associated with poor clinical outcomes of breast cancer patients. **a** The expression of VPS35 in breast cancer tissues and adjacent normal tissues at protein level. Lysates of tumor tissues (T) and matched adjacent noncancerous tissues (N) were analyzed using Western blotting. Eighteen representative pairs are shown. **b** The indicated protein levels in (a) were statistically analyzed. Relative protein expression across all samples. (***p < 0.001; middle). **c** The expression of VPS35 in normal breast epithelia MCF10A and various breast cancer cell lines (MCF‐7, ZR‐75‐1, MDA‐MB-231, Hs578T, SK-BR-3, MDA-MB-453). **d** Representative IHC staining with low expression of VPS35 in breast cancer tissue. **e** Representative IHC staining with high expression of VPS35 in breast cancer tissue. **f** The correlation of VPS35 expression with PFS of breast cancer patients by Kaplan–Meier survival analysis. **g** The correlation of VPS35 expression with OS of breast cancer patients by Kaplan–Meier survival analysis

To illustrate whether VPS35 acts as an oncogenic factor in breast cancer, we investigate the expression level of VPS35 in 52 pairs of breast cancer samples and the adjacent normal samples in the same patients by Western blot. VPS35 was overexpressed in breast cancer clinical samples compared with that in the normal tissues (Fig. 5a, b). Subsequently, we examined VPS35 expression level in a series of breast cancer cell lines, including MCF-7, ZR-75-1, MDA-MB-231, Hs578T, SK-BR-3, MDA-MB-453 and normal breast epithelia MCF‐10A by Western blot (Fig. 5c). VPS35 was upregulated in breast cancer cell lines compared with normal breast epithelia. These data suggested that VPS35 was upregulated in breast cancer tissues and breast cancer cells.

To further elucidate the role of VPS35 in breast cancer development, we analyzed the relationship between VPS35 expression of 120 breast cancer clinical tissues by IHC and clinical pathological factors. There was a significant correlation of VPS35 expression with tumor size (p = 0.01), lymph node metastasis (p = 0.006), and ER negative (p = 0.043), indicating that VPS35 was involved in breast cancer development (Fig. 5d, e and Table 3). To evaluate the prognostic role of VPS35 expression in breast cancer, we conducted Kaplan–Meier survival analysis. VPS35-high patients had significantly lower progression-free survival (PFS) (p = 0.047) and overall survival (OS) (p = 0.029) than VPS35-low patients (Fig. 5f, g). Therefore, the above results suggested that VPS35 might be a significant progressive and prognostic factor in breast cancer.

**Table 3:** Association of VPS35 expression with the clinical pathological characteristics in breast cancers

Factors	Number	VPS35 expression		p value
		Positive	Negative	
Age				
≤ 52	55	33	22	0.73
> 52	65	41	24	
Tumor size (cm)				
≤ 3	63	32	31	0.01*
> 3	57	42	15	
LN metastasis				
Negative	49	23	26	0.006*
Positive	71	51	20	
TNM stage				
I	42	22	20	0.125
II–III	78	52	26	
ER status				
Negative	61	43	18	0.043*
Positive	59	31	28	
PR status				
Negative	59	40	19	0.087
Positive	61	34	27	
HER2 status				
Negative	69	40	29	0.333
Positive	51	34	17	
Molecular subtype				
LuminalA/B	59	30	29	0.499
HER2 amplification	33	18	15	
TNBC	28	18	10	

^*^Indicated statistical significance (*p* < 0.05)

### VPS35 knockdown inhibits breast cancer cell proliferation, migration/invasion and influences autophagy

**Fig. 6:**
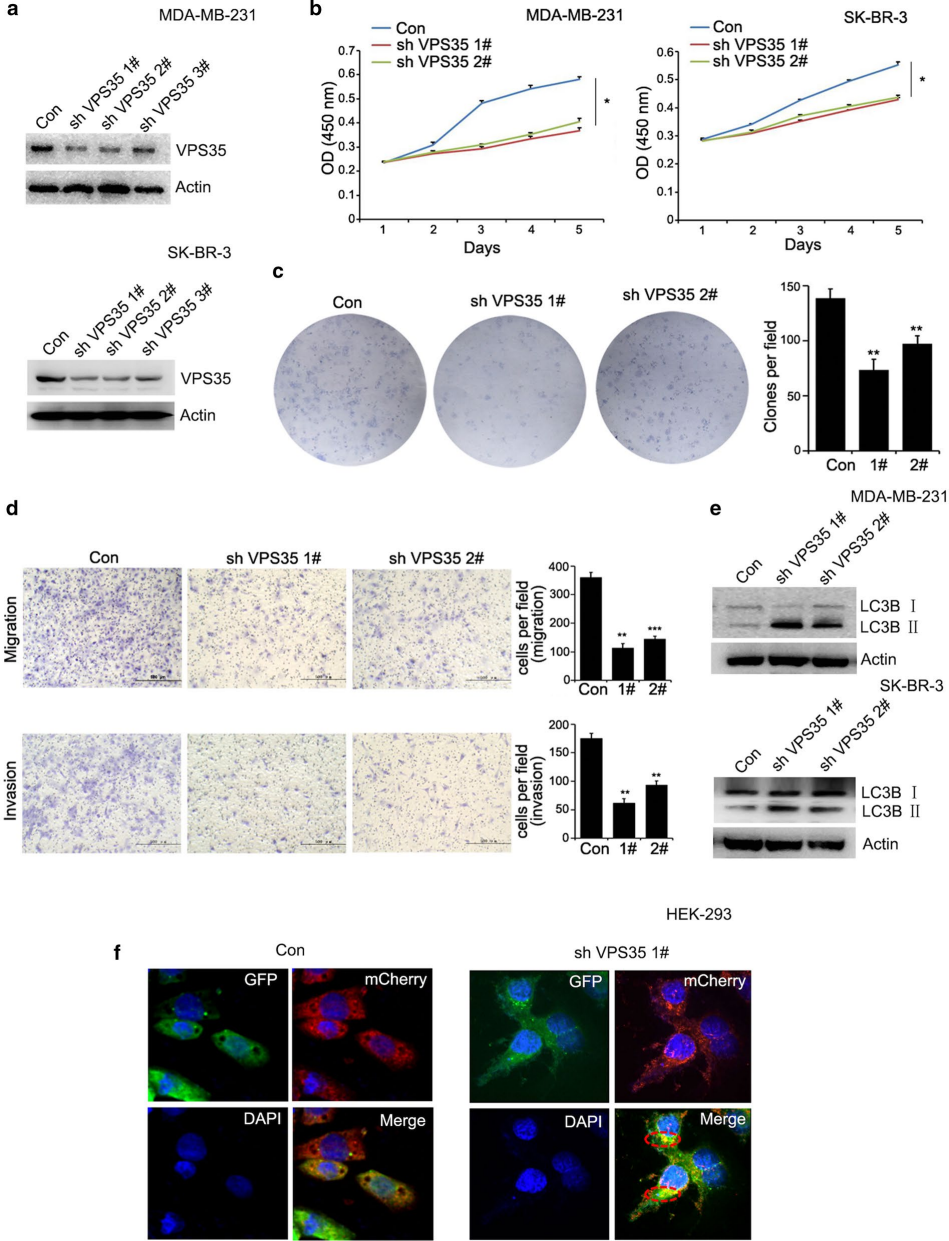
VPS35 knockdown inhibits breast cancer cell proliferation, migration, invasion and induces autophagy. **a** VPS35 expression is substantially suppressed in shVPS35 1#, shVPS35 2#, and shVPS35 3# sublines. **b** CCK8 assay upon VPS35 knockdown in MDA-MB-231 and SK-BR-3. **c** Colony formation assay upon VPS35 knockdown in MDA-MB-231. **d** Transwell migration/invasion assays upon VPS35 knockdown in MDA-MB-231. **e** Knockdown of VPS35 induced the transition of the LC3BI to LC3BII. **f** The track of LC3 expression upon VPS35 knockdown by autophagy flux assay in HEK293. Original magnification was × 600. * *p* < 0.05, ** *p* < 0.01, *** *p* < 0.001

To clarify whether VPS35 is a functional gene in breast cancer cells, we detected cancer cell proliferative abilities by CCK8 and colony formation assays, and cell migrative and invasive capacities by Transwell migration/invasion assays. We constructed VPS35 knockdown (shVPS35) breast cancer cell lines (MDA-MB-231 and SK-BR-3) by three virus-induced VPS35 shRNAs (shVPS35 1#; shVPS35 2#; shVPS35 3#) that target different regions of VPS35 mRNA. The efficiency of VPS35 knockdown was confirmed at protein level (Fig. 6a). We then selected shVPS35 1# and shVPS35 2# for further investigation. The cell growth of breast cancer was significantly inhibited in the shVPS35 1# and shVPS35 2# groups compared with the control group (Fig. 6b). Compared with control group (Con), shVPS35 groups formed less and smaller colonies (Fig. 6c). These suggested that the growing and proliferative abilities clearly reduced on loss of VPS35. One hallmark of cancer characteristics is invasion [16]. Thus, we investigated whether VPS35 knockdown suppressed the migrative and invasive capacities of MDA-MB-231, which is a more aggressive breast cancer cell line, belonging to TNBC. The migration and invasion of breast cancer cells were remarkably decreased upon VPS35 knockdown in MDA-MB-231 (Fig. 6d). Meanwhile, we also conducted proliferation and migration assays on the MCF7 cell line, which belongs to ER^+^ breast cancer. The results showed that the cell growth of MCF7 was decreased upon VPS35 knockdown and the migration and invasion abilities of MCF7 were also inhibited upon loss of VPS35 in the supplementary section (Additional file 1: Figure S1a, b). To further investigate the relationship between VPS35 and autophagy, we found that knockdown of VPS35 induced the transition of the LC3BI to LC3BII in breast cancer cells (Fig. 6e). To track LC3 expression when VPS35 level was silenced, we performed autophagy flux assay. The results showed that yellow LC3 puncta in autophagosomes were increased upon VPS35 knockdown, indicating that autophagosome-lysosome fusion was prevented and the autophagic degradation was blocked by VPS35 silence (Fig. 6f). All these results indicated that VPS35 promotes the progression and aggression of breast cancer and VPS35 plays an essential role in the completion of autophagy process.

## Discussion

In clinical practice, it has made great improvements in survival prognosis of breast cancer patients, but metastasis and recurrence incidences remain to be grown, which were the source of breast cancer mortality. Large-scale researches have demonstrated that autophagy functions as a double-edged sword in cancer development. In line with that, under different circumstances, autophagy is able to act either a pro-survival or pro-death role in breast cancer [17, 18]. Autophagy promotes metastatic breast cancer recurrence through prolonging dormant breast cancer cells survival time [9]. Cytostatic autophagy repressed triple-negative breast cancer cells aggressiveness [10]. Besides, increasing studies suggested autophagy-related encoding genes play crucial roles in progression or inhibition of various cancers, including lung cancer, hepatocellular carcinoma and breast cancer [19–21]. Thus, seeking for potential specific ARGs with prognostic value aroused attention. In this study, we identified the risk model of the 6 ARGs as an independent prognostic factor for breast cancer. So far, among these 6 ARGs, Only TRIM21 and MAP1LC3A, as low-risk autophagy-related genes, have been studied in breast cancer or other cancers. TRIM21 has been demonstrated its anti-oncogenic function in breast cancer and some molecular mechanisms have been revealed. It has been reported that TRIM21 inhibits epithelial-mesenchymal transition (EMT) via Snail ubiquitination in breast cancer cells [22]. In addition, the low expression of TRIM21 indicates worse outcome and promotes cell growth in breast cancer [23]. However, it has not been clarified whether TRIM21 functions its role in breast cancer progression through controlling autophagy. Increasing evidences have elucidated that MAP1LC3A participates in selective autophagy. Impeding this process promotes breast cancer progression [24]. Moreover, the low expression of MAP1LC3A elevates the risk of distant metastasis in triple-negative breast cancer [25].

Among these 6 ARGs, VPS35 was a high-risk factor with prognostic value in breast cancer patients. Our prediction results also showed that the significant correlations of VPS35 with lymph node metastasis, ER/PR negative status, HER2 positive status and triple negative molecular subtype. These above suggested that VPS35 contributes to the aggressiveness of breast cancer.

Vacuolar protein sorting-associated protein 35 (VPS35) is located at 16q11.2, belonging to a group of vacuolar protein sorting (VPS) genes, and acts as component of the retromer cargo-selective complex [26, 27]. VPS35 is defined as an autophagy-related encoding gene owing to as a subunit of retromer participating in the regulation of autophagy process. The retromer is able to sustain lysosome structure stability and normal lysosome function, which participates in regulating autophagy process [28]. Moreover, there is a co-expression network between VPS35 and several proteins involving in autophagy process [29]. More importantly, VPS35 is implicated in both the activity of Wnt signaling pathway and the endocytosis process [30–32]. The endocytosis process is the essential step of the whole autophagy process [33]. It is well known that Wnt signaling pathway is one of stemness-related pathways, which plays a major role in stemness properties acquisition and maintenance in various cancers including breast cancer [34]. CD44, a well-recognized breast cancer stem cell (BCSC) marker, is a well-known target of Wnt/β-catenin signaling and contributes the ‘stemness’ properties to BCSCs [35]. More and more evidence has indicated that autophagy appears to be contribute to the maintenance of stemness properties in BCSC [36, 37]. In BCSCs, autophagy elevates expression of stem cell markers such as CD44 as well as expression of mesenchymal markers such as vimentin [38]. Thus, VPS35 might represent a central regulator in the crosstalk of stemness and autophagy. Based on these, it let us select VPS35 as the target and we speculated that VPS35 may become a key oncogenic factor for the development and progression of breast cancer.

Considerable researches have demonstrated that VPS35 plays an important role in Parkinson’s disease [39, 40]. Only one study showed that VPS35 promoted the proliferation of hepatoma cells through the PI3K/AKT signaling pathway [41]. However, little is known regarding the expression status, clinical and prognostic significance, and functional role of VPS35 in breast cancer and other cancers.

Therefore, we firstly investigated the role of VPS35 in breast cancer. In our present study, we found that VPS35 was high level in breast cancer tissues compared with normal breast tissues. Consistent with the prediction results, VPS35 was positively correlated with lymph node metastasis and ER negative, indicating that VPS35 acts as an oncogenic factor in breast cancer development. Further analyses were performed to clarify the biological function of VPS35 in breast cancer. Our findings illustrated that VPS35 promoted cell proliferative ability by clonal formation assay and VPS35 accelerated cell migration and invasion capacities by Transwell migration/invasion assays, suggesting that VPS35 is involved in the malignant process of breast cancer. Nevertheless, how VPS35 influencing the aggressiveness of breast cancer provokes our thought. Subsequently, we analyzed the possible molecular mechanism of VPS35 promoting breast cancer progression. Our findings elucidated that VPS35 knockdown induced the transition of the LC3BI to LC3BII in breast cancer cells and yellow LC3 puncta in autophagosomes increasing. It might result from that retromer deficiency by VPS35 silencing impaired the lysosome function and inhibited autophagosomes degradation of the final stage of autophagy process, as well as reflectively enhanced autophagy initiation, which all eventually induced that the level of LC3-II existing on the liminal membrane of autophagosome was accumulated and elevated [28, 42, 43]. The above all suggested that VPS35 knockdown impeded the completion of autophagy process and VPS35 is essential element for autophagy accomplishment in breast cancer cells. Thus, VPS35 might increase the proliferative and invasive abilities of breast cancer cells mediating by autophagy regulation. And we speculated that VPS35 also might participate in regulating stemness properties of BCSCs through controlling autophagy process in breast cancer. Based on the above, in-depth study of VPS35 in breast cancer is demanded to confirm the molecular regulation mechanism.

## Conclusion

In conclusion, we identified a novel autophagy-related prognostic risk model consisting of six encoding gene (VPS35, TRIM21, PRKAB2, RUFY4, MAP1LC3A and LARP1) in breast cancer. It is our novel finding that VPS35, as an autophagy-related encoding gene, is upregulated in breast cancer and positively associated with lymph node metastasis and ER negative. VPS35 is a necessary element for autophagy completion and also promotes breast cancer cell proliferation, migration and invasion. Hence, VPS35 may serve as a promising novel oncogenic factor, prognostic biomarker and therapeutic target for breast cancer.

## Supplementary Information

**Additional file 1: Figure S1.** VPS35 knockdown inhibits breast cancer cell proliferation, migration and invasion in ER^+^ breast cancer. **a** CCK8 assay upon VPS35 knockdown in MCF-7. **b** Transwell migration/invasion assays upon VPS35 knockdown in MCF-7. * *p* < 0.05, ** *p* < 0.01.

## Data Availability

All data utilized in this study are included in this article and all data supporting the findings of this study are available on reasonable request from the corresponding author.

## Abbreviations

ARGs: Autophagy-related encoding genes
VPS35: Vacuolar protein sorting-associated protein 35
TCGA: The Cancer Genome Atlas
GSEA: Gene Set Enrichment Analysis
HR: Hazard ratio
ROC: Receiver operating characteristics
ER: Estrogen receptor
PR: Progesterone receptor
HER2: ERBB2 receptor
OS: Overall survival
PFS: Progression-free survival
BCSC: Breast cancer stem cell
