# Supplementary figures and images for: A novel autophagy-related genes prognostic risk model and validation of autophagy-related oncogene VPS35 in breast cancer
Source: Cancer Cell Int. 2021 May 17;21:265. doi: 10.1186/s12935-021-01970-4 (PMC8130280; doi:10.1186/s12935-021-01970-4)

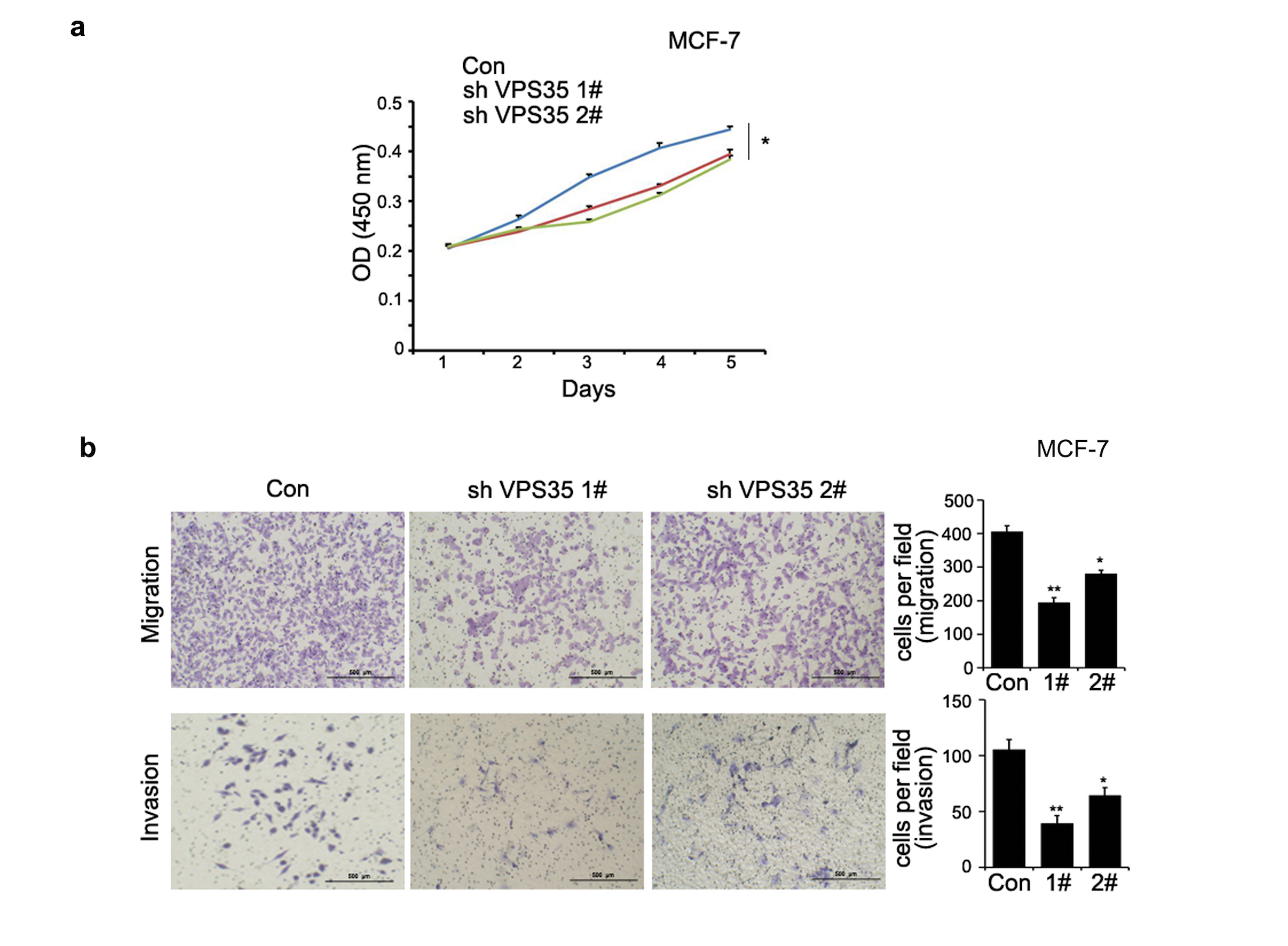

Supplement: Supplementary file 1 — Additional file 1: Figure S1. VPS35 knockdown inhibits breast cancer cell proliferation, migration and invasion in ER+ breast cancer. a CCK8 assay upon VPS35 knockdown in MCF-7. b Transwell migration/invasion assays upon VPS35 knockdown in MCF-7. * p < 0.05, ** p < 0.01. [file 12935_2021_1970_MOESM1_ESM.jpg]
